# Heterospecific alarm-call recognition in two warbler hosts of common cuckoos

**DOI:** 10.1007/s10071-019-01307-9

**Published:** 2019-09-10

**Authors:** Jiangping Yu, Hailin Lu, Wei Sun, Wei Liang, Haitao Wang, Anders Pape Møller

**Affiliations:** 1 Jilin Engineering Laboratory for Avian Ecology and Conservation Genetics, School of Life Sciences, Northeast Normal University, Changchun, 130024 China; 2 Ministry of Education Key Laboratory of Vegetation Ecology, School of Life Sciences, Northeast Normal University, Changchun, 130024 China; 3 Jilin Provincial Key Laboratory of Animal Resource Conservation and Utilization, School of Life Sciences, Northeast Normal University, Changchun, 130024 China; 4 Ministry of Education Key Laboratory for Ecology of Tropical Islands, College of Life Sciences, Hainan Normal University, Haikou, 571158 China; 5 Ecologie Systématique Evolution, Université Paris-Sud, CNRS, AgroParisTech, Université Paris-Saclay, 91405 Orsay Cedex, France; 6 Ministry of Education Key Laboratory for Biodiversity Science and Ecological Engineering, College of Life Sciences, Beijing Normal University, Beijing, 100875 China

**Keywords:** Alarm call, Brood parasitism, Heterospecific recognition, Playback

## Abstract

Species facing similar selection pressures should recognize heterospecific alarm signals. However, no study has so far examined heterospecific alarm-call recognition in response to parasitism by cuckoos. In this study, we tested whether two sympatric host species of the common cuckoo *Cuculus canorus*, Oriental reed warbler *Acrocephalus orientalis* (ORW, main host), and black-browed reed warbler *Acrocephalus bistrigiceps* (BRW, rare host), could recognize each other’s alarm calls in response to cuckoos. Dummies of common cuckoo (parasite) and Eurasian sparrowhawk *Accipiter nisus* (predator) were used to induce and record alarm calls of the two warbler species, respectively. In the conspecific alarm-call playback experiments, ORW responded more strongly to cuckoo alarm calls than to sparrowhawk alarm calls, while BRW responded less strongly to cuckoo alarm calls than to sparrowhawk alarm calls. In the heterospecific alarm-call playback experiments, both ORW and BRW responded less strongly to cuckoo alarm calls than sparrowhawk alarm calls. BRW seemed to learn the association between parasite-related alarm calls of the ORW and the cuckoo by observing the process of ORW attacking cuckoos. In contrast, alarm calls of BRW to cuckoos were rarely recorded in most cases. BRW with low parasite pressure still developed recognition of heterospecific parasite-related alarm call. Unintended receivers in the same community should recognize heterospecific alarm calls precisely to extract valuable information.

**Electronic supplementary material:**

The online version of this article (10.1007/s10071-019-01307-9) contains supplementary material, which is available to authorized users.

## Introduction

Acoustic signals play an important role in animal communication systems. These signals can transfer diverse and meaningful information from a signaller to a receiver (e.g., Clay and Zuberbühler 2011; Clay et al. 2012; Suzuki and Kutsukake 2017) and influence the receiver’s behavior and physiology (Endler 1993; Bradbury and Vehrencamp 2011). Previous studies showed that many vertebrates evolved alarm calls to warn others of danger (e.g., Seyfarth et al. 1980; Macedonia and Evans 1993; Fichtel and Kappeler 2002; Price and Fischer 2014). They could encode information about the perceived threat in their alarm calls by call types, calling rates, duration of calls, compositional syntax, and other characteristics of calls (e.g., Manser et al. 2002; Fichtel 2004; Templeton et al. 2005; Suzuki et al. 2016). Interestingly, there are still large differences in the characteristics and information encoding method of alarm calls among species. Such heterospecific alarm-call recognition is widespread in animal communities.

Predation has been considered the main driving force behind the evolution of alarm calls (Gill and Bierema 2013; Wheatcroft and Price 2015). Prey can produce alarm calls to convey predator-related information to potential receivers (e.g., Templeton et al. 2005; Griesser 2008; Soard and Ritchison 2009). Species belonging to the same community may face similar predator pressure, and they avoid predation by recognizing conspecific and heterospecific alarm signals (Templeton and Greene 2007; Hetrick and Sieving 2011; Bshary and Noë 1997; Magrath et al. 2015). For example, sympatric redfronted lemurs (*Eulemur fulvus rufus*) and Verreaux’s sifakas (*Propithecus verreauxi verreauxi*) recognized each other’s alarm calls and reacted appropriately to each other’s aerial or general alarm calls (Fichtel 2004).

Brood parasitism is another important driving force behind avian alarm calls. In the avian kingdom, some taxa such as cuckoos lay eggs in the nests of other bird species (hosts) and transfer parental care and its cost to hosts (Davies 2000; Soler 2014). However, brood parasites appear to be specialized in their host use (regular hosts), and some species are immune to brood parasitism (rare hosts or non-hosts) (Wyllie 1981; Payne 1997; Davies 2000; Langmore et al. 2005; Yang et al. 2012). Thus, species belonging to the same community may face different intensities of selection from brood parasites. The question then arises as to whether common hosts, rare hosts, or non-hosts recognize alarm calls of each other in response to parasites?

Regular hosts have evolved the ability to recognize parasites (Welbergen and Davies 2008; Feeney et al. 2013; Ma et al. 2018a, b). In addition, hosts could produce alarm calls to transmit parasite-related information to conspecifics (Gill and Sealy 2004; Welbergen and Davies 2008; Wheatcroft and Price 2015). For example, reed warblers (*Acrocephalus scirpaceus*) recruit peers to chase away brood parasites and enhance their nest defense using alarm calls (Welbergen and Davies 2008; Campobello and Sealy 2011). While few rare hosts or non-hosts performed similar attack behavior on brood parasites, which look alike, they mistake parasites for predators (cuckoo–hawk mimicry, Davies and Welbergen 2008; Trnka et al. 2015; but see Ma et al. 2018a). However, Yu et al. (2017a) showed that even rare host great tits (*Parus major*) could distinguish parasites, because tits produced different alarm calls in response to Eurasian sparrowhawks *Accipiter nisus* and common cuckoos (*Cuculus canorus*). These calls caused different response behaviors to conspecific receivers. Alarm calls are widely used in several contexts. Reception and precise recognition of other species’ alarm calls is necessary for community members. Even intended receivers of parasite-related alarm calls might be a restricted subset of community members (e.g., Welbergen and Davies 2008; Feeney et al. 2013; Wheatcroft and Price 2015), and still, it could not be ruled out that other community members (unintended receivers) recognize parasite-related alarm calls.

General theory demonstrates that interactions between signal producers and signal receivers should influence the evolution of signal recognition (Guilford and Dawkins 1991; Johnstone 1997). Wheatcroft and Price (2015) suggested that variation in the suite of receivers is a powerful force affecting signal evolution, as predator-related alarm calls evolved faster than parasite-related alarm calls in *Phylloscopus* species. However, few studies have tested heterospecific alarm-call recognition of species under different intensity of selection. In this study, we first examined whether regular host oriental reed warblers (*Acrocephalus orientalis*) (hereafter ORWs) and rare host black-browed reed warblers (*Acrocephalus bistrigiceps*) (hereafter BRWs) have the ability to distinguish between common cuckoos and sparrowhawks using similar methods as Yu et al. (2017a), and restrict their response behavior to conspecifics alarm calls. Heterospecific alarm-call recognition has been demonstrated experimentally through playback studies in several species (e.g. Nuechterlein 1981; Sullivan 1984), and thus, we further tested whether ORWs and BRWs could recognize alarm calls of each others to parasites by conspecific and heterospecific playback experiments. If signal recognition evolves in isolation between predator pressure and brood parasite pressure, we predicted that ORWs and BRWs would recognize and appropriately respond to alarm calls of each other to the predator, but would not recognize each others’ alarm calls to the parasite.

## Materials and methods

### Study species and study area

ORWs and BRWs both build open cup-shaped nests in the same type of habitats (reed swamps). ORWs are regular hosts of the common cuckoo and have evolved aggressive behavior towards cuckoos (Yang et al. 2014; Li et al. 2015). BRWs are rarely used as a host (rare host) with a very low parasitism rate (Yang et al. 2017), and they occasionally behave aggressively towards cuckoos (see “Results”). The two sympatric species ORW and BRW are vulnerable to many of the same predators, while parasitism rate of the two species by the common cuckoo differed significantly, since the rate of parasitism was much higher in the ORW than in the BRW (Yang et al. 2017). Thus, the common cuckoo, ORW and BRW system, provides an ideal model system for testing whether they recognize each other’s alarm calls under different levels of brood parasitism.

This study was performed in Zhalong National Nature Reserve (46°48ʹ‒47°31ʹN, 123°51ʹ‒124°37ʹE) located on the northern Songnen Plain in Heilongjiang Province, northeast China during the breeding seasons (June–August) 2016–2017. We searched for nests of the two species every 3–5 days and monitored the activities of parents to confirm their reproductive stage (Li et al. 2015; Yang et al. 2015).

### Dummy experiment

During June–July 2016, we randomly presented taxidermic dummies of a common cuckoo (nest parasite) and a sparrowhawk (predator) to ORW and BRW (each nest received two dummy presentations in random order) during the incubation period. Two specimens per species were used to reduce pseudo-replication (e.g., Davies and Welbergen 2008). Each specimen posed as naturally standing with wings naturally closed and attached to a bamboo stick. Because ORW usually performed intense attack behavior towards the common cuckoo specimen, we kept all specimens in a small cage (28 × 22 × 25 cm) made from thin green wire to protect specimens from damage (Davies and Welbergen 2009; Li et al. 2015). The function of a control stimulus is to act as a criterion of behavioral responses in target birds. We have studied ORWs and BRWs in our study area for many years (from 2012 to date, e.g., Yang et al. 2015, 2016, 2017), and conducted several dummy experiments (common cuckoo, sparrowhawk, Oriental turtle dove *Streptopelia orientalis*, etc., unpublished data). We can exclude the possibility that target ORWs and BRWs responded similarly to all birds near the nest. Thus, we think that no control dummy in this paper is sufficient to explain our predictions. When focal parent birds were absent, one person (assistant, Y. Y.) placed one specimen at a distance of 0.5 m from the focal nest and at a height of 0.5 m above the nest rim with the head facing the rim (Li et al. 2015) and left quickly. The researcher (H. L.) remained at hiding spots about 5–6 m from the nest to record behavior and alarm calls. Recording of alarm calls and behavior started when parent birds were observed within approximately 2 m of the specimen. Each recording lasted 5 min (Davies and Welbergen 2008; Yu et al. 2017a). We recorded the dummy response of ORW and BRW as (1) the number of attacks of focal parent birds (we counted exactly the number of attacks indoor by playing back the video); and (2) the maximum number of ORW and BRW being attracted during experiments (the maximum number of simultaneously observed warblers). A trial was terminated if no parent bird arrived within 15 min, and the next trial started at least 1 h later. The trials were conducted during sunny days between 0730–1100 and 1430–1800, using a TASCAM DR-44WL portable digital recorder (TEAC Corporation, Tokyo, Japan) and a Sennheiser MKH 416 P 48 U external directional microphone (Sennheiser electronic GmbH & Co. KG, Wedemark, Germany). Sampling frequency was set at 44.1 kHz and sampling resolution was 24 bits. Video recorders were set up at a distance of 2 m from the nest to record the experimental process.

### Playback experiments

During June–July 2017, we conducted playback experiments with ORW and BRW during their incubation period. Alarm calls for playback were those of warblers to common cuckoo specimens (referred to as “ORW cuckoo alarm calls” and “BRW cuckoo alarm calls”) and sparrowhawk specimens (referred to as “ORW hawk alarm calls” and “BRW hawk alarm calls”). All recorded in 2016. A total of 12 records of ORW alarm calls from six nests were used (six cuckoo alarm and six hawk alarm) to avoid or reduce pseudo-replication (Kroodsma 1989). Meanwhile, a total of 14 records of BRW alarm calls from seven nests were used (seven cuckoo alarm and seven hawk alarm). In this study, we chose the background noise as the control stimulus (from four selected alarm-call records of ORW and BRW, respectively). Thus, we not only played back a set of conspecific alarm calls to warblers, but also played back another set of heterospecific alarm calls to them. Background noise used as a control stimulus allowed us to assess the standard response behavior in warblers to different alarm calls without any playback of the call (vocal production in birds is usually functional, such as repelling a competitor, group cohesion and announcing the presence of food), ensuring that warblers are not responding to the properties of background sounds in recordings. We selected alarm calls of high quality and removed low-frequency noise (< 0.2 kHz) from these recordings (Yu et al. 2016). When recordings had overlapping calls, we deleted them. We tried our best not to change the call types and calling rates of the stimuli (Yu et al. 2016). Avisoft SASLab Pro 5.2 software (Avisoft Bioacoustics, Glienicke, Germany) was used to construct the playback stimuli.

Before the experiments started, we confirmed that focal parent birds were absent (no birds were visible around the nest and no birds were calling within 2 min). A speaker attached to a bamboo stick was placed at a distance of 1 m from the focal nest. The duration of each experiment was 7 min, including 2 min playback, and 5 min of subsequent observations. Behavioral responses during 7 min were recorded, included response latency (when responses occurred) and response duration (duration from the onset of behavioral response to termination) (Yu et al. 2016). Video recorders were set up to record the experimental process. Alarm calls of ORW were played back to ORW (referred to as “O-O playback” hereafter), and alarm calls of BRW were played back to BRW (referred to as “B-B playback” hereafter). Considering that birds could recognize threat information from conspecific and heterospecific alarm calls, alarm calls of ORW and BRW heterospecific playback were conducted (ORW’s alarm calls played back to BRW was referred to as “O-B playback” hereafter and BRW’s alarm calls played back to ORW was referred to as “B-O playback” hereafter) to investigate whether ORW and BRW could recognize each others’ alarm calls. The researcher (H. L.) remained motionless 5 m away to score response latency and response duration using a digital stopwatch. In addition, all behavioral responses were recorded by H. L. to avoid variation among observers.

Playback experiments were carried out in clear and windless weather with at least 1 h intervals, and one set of conspecific or heterospecific alarm calls (three playback stimuli for each set) for one nest was finished within 1 day (0730–1100 and 1430–1800). Each stimulus was played at the same volume and the sound pressure level at 1 m ≈ 75 dB for all trials. Alarm calls used for playback and playback order were determined using random assignment to reduce the possibility that individuals encountering calls produced by themselves. If two sets of playbacks were conducted (alarm calls of conspecific and heterospecific) at a same nest, the experimental interval was at least 2 days, and the conspecific alarm-call playback was broadcast first (two nests for “B-O playback” and four nests for “O-B playback”). There should be no carryover effects from the first (conspecific) playback due to the time between playbacks. During playback experiments, the observer (H. L.) was blind with respect to the playback order, since that was determined by the assistant (Y. Y.).

### Statistical methods

All data in this study were analyzed using R 3.4.3 software (http://www.r-project.org). For the response variables during dummy experiments, generalized linear mixed models (GLMMs, glmer in R package lme4) with a Poisson error structure and log-link function were used for the number of attacks of focal parent birds and the maximum number of warblers being attracted. For the response variables during three categories of playback experiments, we used GLMMs with a Poisson error structure and log-link function for analyses of response latency and duration. In the event of a significant effect of treatment, we further performed post hoc pairwise comparison between treatments. Because two-group comparison after multiple comparisons will increase the probability of type I errors, we used Bonferroni correction to adjust *P* values (P.adjust function in R package stats, Yu et al. 2017b). We calculated *P* values for all models using Wald Chi-square tests with the Anova function in the car package. For all models, treatment was treated as a fixed term and individuals distinguishing birds’ nests and trial order as random terms. In addition, Mann–Whitney *U* tests were performed to evaluate differences in the response latency and duration of warblers to parasite-related or predator-related alarm call between the conspecific and heterospecific playbacks. All tests were two-tailed, and the significance level was set to 0.05. Mean ± SE are presented.

## Results

### Dummy experiment

**Fig. 1:**
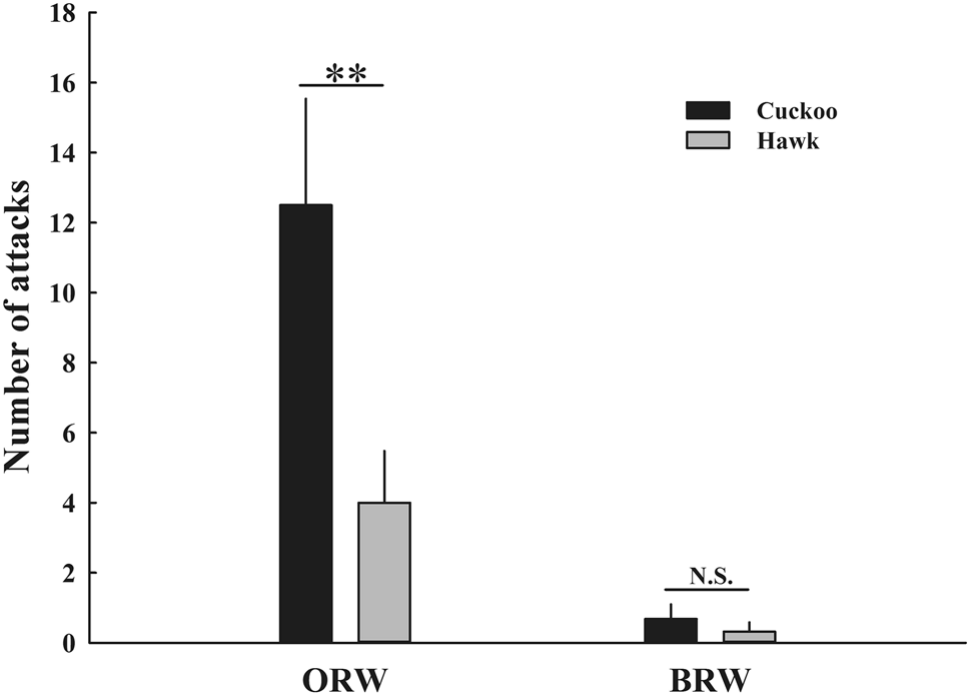
The number of attacks of focal ORW and BRW parent birds in response to presentations of cuckoo and hawk specimens (mean ± SE). ^N.S.^*P* > 0.05; **P < 0.01

The number of attacks of focal warbler parent birds to stimuli by two host species differed significantly in ORW, but not in BRW. ORW attacked cuckoos significantly more strongly than hawks ($\chi_{1}^{2}$ = 66.55, *n* = 18, *P *< 0.001, Fig. 1). In contrast, BRW rarely attacked cuckoo and hawk ($\chi_{1}^{2}$ = 2.82, *n* = 19, *P *= 0.09, Fig. 1). There was no significant difference in the maximum number of warblers being attracted ($\chi_{1}^{2}$ = 0.30, *P* = 0.58 for ORWs and $\chi_{1}^{2}$ = 0.00, *P* = 1.00 for BRWs).

### Playback experiment

**Fig. 2:**
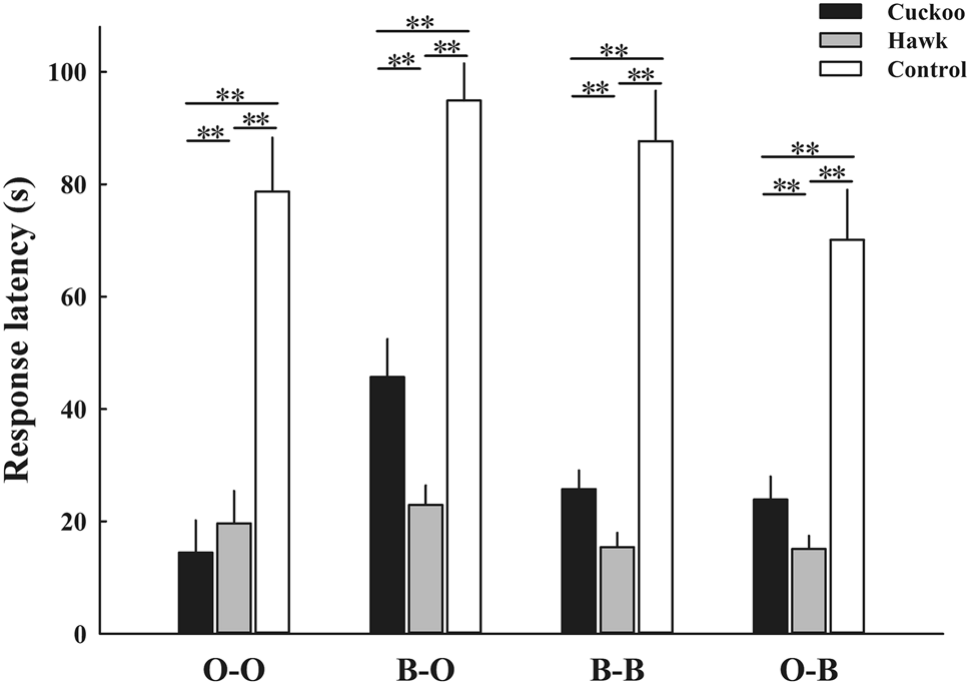
Response latency of ORWs and BRWs to control (background noise), and cuckoo and hawk alarm calls in three categories of playback experiments (mean ± SE). O–O refers to the alarm calls of ORW when played back to ORW. B–O refers to the alarm calls of BRW when played back to ORW. B–B refers to the alarm calls of BRW when played back to BRW. O–B refers to the alarm calls of ORW when played back to BRW. **P < 0.01

**Fig. 3:**
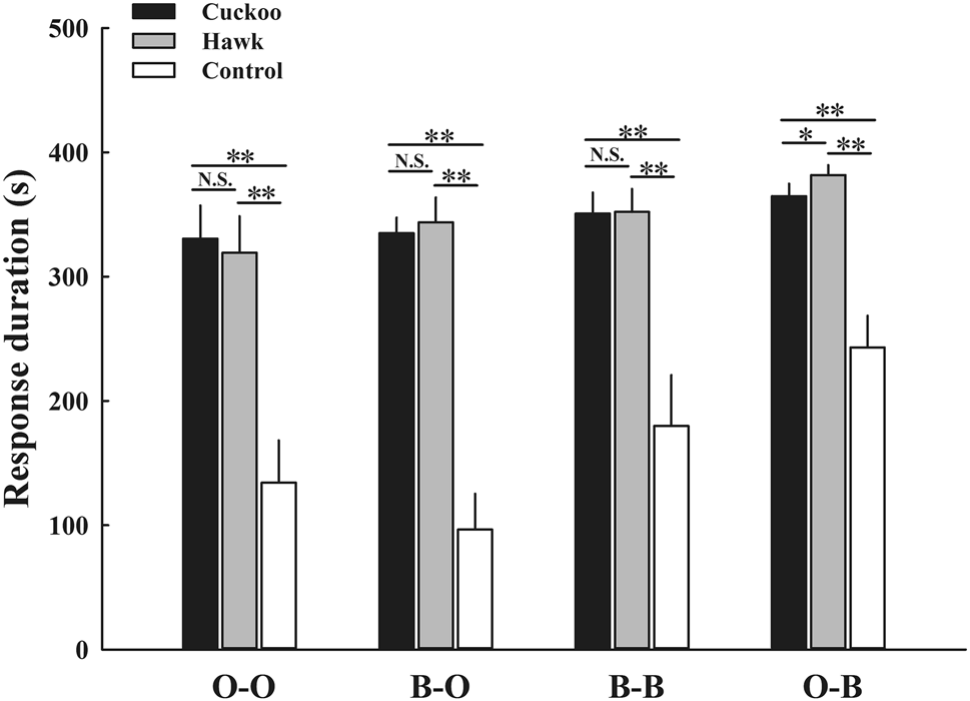
Response duration (mean ± SE) of ORWs and BRWs to control (background noise), and cuckoo and hawk alarm calls in three categories of playback experiments (mean ± SE). O–O refers to the alarm calls of ORW when played back to ORW. B–O refers to the alarm calls of BRW when played back to ORW. B–B refers to the alarm calls of BRW when played back to BRW. O–B refers to the alarm calls of ORW when played back to BRW. ^N.S.^*P* > 0.05; *P < 0.05; **P < 0.01

The response latency and response duration of ORWs and BRWs to three playback stimuli differed significantly between conspecific and heterospecific alarm calls (O–O/B–O/B–B/O–B) from playback experiments (*P *< 0.001 for all multiple comparisons, Table 1). Differences in response variables between control (background noise) and the two alarm calls were all significant (Table 1, Figs. 2, 3).

**Table 1:** Results of post hoc comparisons for response latency and response duration of ORWs and BRWs to control (background noise), cuckoo, and hawk alarm calls in three categories of playback experiments

Behavioral parameter	$\chi_{2}^{2}$	*P*		Post hoc *P*	
				Hawk	Cuckoo
O-O (*n* = 20)					
Response latency	543.48	< 0.001**	Cuckoo	< 0.001**	
			Control	< 0.001**	< 0.001**
Response duration	1242.80	< 0.001**	Cuckoo	0.08	
			Control	< 0.001**	< 0.001**
O-B (*n* = 19)					
Response latency	575.06	< 0.001**	Cuckoo	< 0.001**	
			Control	< 0.001**	< 0.001**
Response duration	536.95	< 0.001**	Cuckoo	0.02*	
			Control	< 0.001**	< 0.001**
B-B (*n* = 19)					
Response latency	682.89	< 0.001**	Cuckoo	< 0.001**	
			Control	< 0.001**	< 0.001**
Response duration	911.74	< 0.001**	Cuckoo	1.00	
			Control	< 0.001**	< 0.001**
B-O (*n* = 20)					
Response latency	359.70	< 0.001**	Cuckoo	< 0.001**	
			Control	< 0.001**	< 0.001**
Response duration	685.47	< 0.001**	Cuckoo	1.00	
			Control	< 0.001**	< 0.001**

Results are from generalized linear mixed models; treatment was treated as a fixed term and individuals distinguishing birds’ nests and trial order as random terms. *P* values were adjusted by Bonferroni correction

*P < 0.05; **P < 0.01

In “O-O playback” experiments, the response latency of ORWs to conspecific cuckoo alarm calls was significantly shorter than those to hawk alarm calls (Table 1, Fig. 2), while the response duration to cuckoo and hawk alarm calls was similar (Table 1). In “B-B playback” experiments, the response latency of BRWs to conspecific cuckoo alarm calls was significantly longer than those to hawk alarm calls (Table 1, Fig. 2), while the response duration to cuckoo and hawk alarm calls was similar (Table 1).

In “B-O playback” and “O-B playback” experiments, both ORWs and BRWs had significantly longer latency to response to heterospecific cuckoo alarm calls than to heterospecific hawk alarm calls (Table 1, Fig. 2). The response duration of ORWs to heterospecific cuckoo and hawk alarm calls was similar (Table 1). The response duration of BRWs to heterospecific cuckoo alarm calls was significantly shorter than to heterospecific hawk alarm calls (Table 1, Fig. 3).

There was no difference between the response latency and duration of ORWs and BRWs to conspecific and heterospecific hawk alarm calls and to conspecific and heterospecific cuckoo alarm calls (*P* > 0.58 for all tests), except for the response latency of ORWs to heterospecific cuckoo alarm calls was significantly longer than to conspecific cuckoo alarm calls (Mann–Whitney *U* test, *W* = 49.00, *P *< 0.001).

## Discussion

In our dummy experiments, ORWs more intensely attacked cuckoos than sparrowhawks, while BRWs did not perform attacks. Birds could adopt corresponding behavioral strategies to protect their nests or themselves by assessing and trading threat categories of different intruders (Mahr et al. 2015). The cuckoo as a brood parasite poses less of a threat to parent birds, but a larger threat to their current reproductive investment. Violently attacking cuckoos is a common and suitable defense strategy of regular hosts to prevent parasitism (Molnár 1944; Wyllie 1981; Davies and Brooke 1988; Welbergen and Davies 2008; Li et al. 2015), but rare hosts do not have to do so. The sparrowhawk as a predator mainly threatens adults and vulnerable fledglings. When birds detect perched raptors during the breeding season, one strategy that they could adopt is driving raptors off (Wheatcroft and Price 2013), but it entails costs in terms of time and energy expenditure and injury or death caused by predators. Another strategy that birds could take is not to respond aggressively to predators if they are unlikely to threaten their offspring (Montgomerie and Weatherhead 1988). We conducted our experiments during the incubation period, and sparrowhawks never take eggs. Thus, we suggest that both ORW and BRW adopted the latter strategy to avoid the risk of injury by the predator, as most of them tended to retreat and remain 2–7 m away from the sparrowhawk mounts, often jumping from one side to another. Our results supported previous studies, showing that regular hosts could distinguish between cuckoo and sparrowhawk, demonstrating strongly aggressive behavior against cuckoos (e.g., Yang et al. 2014; Li et al. 2015; Liang and Møller 2015).

In playback experiments with conspecific alarm calls (O-O and B-B), ORWs returned to their breeding territories significantly more quickly in response to cuckoo alarm calls than to hawk alarm calls, while BRWs returned significantly slower to cuckoo alarm calls than to hawk alarm calls. The whole process of parasites laying one egg into the host’s nest was very fast (only a few seconds, Wang et al.’s personal observation). Regular hosts could increase the possibility of preventing a parasitism event by producing additional anti-parasite behavior if they responded quickly when gaining parasite-related information (Gill and Sealy 2004; Welbergen and Davies 2008; Feeney et al. 2013; Wheatcroft and Price 2015). For example, yellow warblers (*Dendroica petechia*) respond to their parasite-related “seet” calls quickly and females rush to sit on their nests to avoid parasitism (Gill and Sealy 2004). Rare hosts or non-hosts do not need to make any response behavior quickly as parasitism events were rare. For sparrowhawk alarm calls, warblers generally had relative short response latency (Fig. 2). Fast response to conspecific alarm calls will help prey to reduce predation risk, because they could adopt appropriate anti-predatory behavior after identifying and locating a stationary predator (Cunningham and Magrath 2017; Méndez et al. 2017). In addition, ORWs and BRWs had similar response duration to conspecific cuckoo and hawk alarm calls, suggesting that warblers might need time to ensure that there is no threat in the surroundings when they receive threat information. As ORWs and BRWs behave differently in response to latency to conspecific cuckoo and hawk alarm calls, we suggest that alarm calls of warblers could encode and convey different information to conspecifics, and rare host BRWs could discriminate the cuckoo from the sparrowhawk. However, we did not examine and confirm the rules of information encoded in ORW and BRW alarm calls in this study, although that could be explored in future research.

In heterospecific alarm-call playback experiments (B–O and O–B), both ORW and BRW response latency to heterospecific cuckoo alarm calls were significantly longer than that to heterospecific hawk alarm calls. Previous studies have shown that a large amount of information could be encoded in alarm calls of birds, such as the type and the degree of threats (Templeton et al. 2005; Courter and Ritchison 2010; Sieving et al. 2010). Only if signal receivers recognize different information from alarm calls, and the signal is relevant and provides reliable information for them (Goodale and Ruxton 2010; Magrath et al. 2015), could they adopt appropriate response behavior. ORWs responded in the same way to BRWs’ hawk alarm calls as to conspecific hawk alarm calls, indicating that they could recognize heterospecific predator-related alarm calls. ORWs had significantly longer response latency to heterospecific cuckoo alarm calls than to conspecific cuckoo alarm calls. Thus, we suggest that ORWs could recognize threat information from BRW alarm calls, but not recognize the calls as indicative of presence of a cuckoo, because, if ORWs receive parasite-related information, they should respond quickly. The response of BRWs to heterospecific alarm calls was consistent with that against conspecific alarm calls. Alarm calls of ORWs should contain threat type and threat level information about intruders (see above). BRWs not only responded appropriately to heterospecific predator-related alarm calls, but also heterospecific parasite-related alarm calls. Although ORW cuckoo alarm calls caused the strongest response behavior of conspecific individuals, BRWs performed lower levels of response to them. These results indicated that BRWs could precisely recognize ORW alarm calls, including cuckoo alarm calls.

Our results supported the prediction that ORW and BRW could share predator-related alarm information. However, BRW with low parasite pressure could still develop heterospecific recognition of parasite-related alarm calls. The acquisition of heterospecific alarm-call responses might evolve via social learning by associating calls in particular contexts (Shriner 1999; Davies and Welbergen 2009; Feeney and Langmore 2013). Aggressive displays and alarm calls emitted by hosts should attract intended receivers (e.g., Welbergen and Davies 2008; Feeney et al. 2013), which are also likely to attract unintended receivers. Alarm calls of ORW to common cuckoos were reliable for BRWs, because the probability of regular host ORW encountered and attacked common cuckoo was very high in our study area. Thus, it did not cause a strong response behavior of BRWs. Here, we suggest that BRWs could learn the association between parasite-related alarm calls of ORWs and the cuckoo by observing the process of ORWs attacking cuckoos, and assess these alarm calls as less relevant. In contrast, rare host BRWs only interacted little with the common cuckoo. They most likely did not need to encode the threat information about parasites in their alarm calls. Even if BRWs could encode parasite-related information, it is rarely possible for ORWs to hear alarm calls of BRWs in response to cuckoos in most cases. Thus, alarm calls of BRWs to a cuckoo may not be reliable or pertinent in the same way as ORW. BRWs performed aggressive behavior and produced alarm calls to predators with high probability, and ORWs could recognize BRW alarm calls, which are produced in response to predators.

In mixed-species flocks, recognition of heterospecific alarm signals is thought to benefit receivers by gaining access to an additional source of information to avoid errors and subsequent risk (Bshary and Noë 1997; Sridhar et al. 2009; Magrath et al. 2015). That occurs typically in closely related species (Nuechterlein 1981; Seyfarth and Cheney 1990; Fichtel 2004), such as sympatric sister species ORW and BRW in our study area. However, variable signal information and function (e.g., false alarms, Munn 1986; Ridley and Child 2009) makes such alarm-call systems complex. Reliability and consistency of alarm signals are also important for allowing receivers to adopt response behaviors (Goodale and Ruxton 2010; Magrath et al. 2015). Thus, regular host ORWs responded slightly to alarm calls of rare host BRWs to a parasite. For unintended receivers in the same community, they should recognize heterospecific alarm calls precisely to extract valuable information. Consistently, BRW distinguishes ORWs’ relative predator-related alarm calls from irrelative parasite-related alarm calls. Studies of the recognition of heterospecific alarm calls under different selection pressures will enhance our understanding of the evolution of signal recognition.

## Electronic supplementary material

Below is the link to the electronic supplementary material.

Supplementary material 1 (XLS 48 kb)
